# *In vitro* antimicrobial susceptibility of *Mycoplasma pneumoniae* isolates across different regions of China in 2023

**DOI:** 10.1093/jacamr/dlaf124

**Published:** 2025-07-30

**Authors:** Chao Yan, Yujie Chen, Xue Ren, Xinyu Jia, Bing Du, Hanqing Zhao, Yanling Feng, Guanhua Xue, Jinghua Cui, Xuanfeng Liu, Jing Yuan

**Affiliations:** Department of Bacteriology, Capital Institute of Pediatrics, Beijing, China; Department of Bacteriology, Capital Institute of Pediatrics, Beijing, China; Department of Bacteriology, Capital Institute of Pediatrics, Beijing, China; Capital Institute of Pediatrics, Chinese Academy of Medical Sciences & Peking Union Medical College, Beijing, China; Department of Bacteriology, Capital Institute of Pediatrics, Beijing, China; Department of Allergy, Capital Center for Children’s Health, Capital Medical University, Beijing, China; Department of Bacteriology, Capital Institute of Pediatrics, Beijing, China; Department of Bacteriology, Capital Institute of Pediatrics, Beijing, China; Department of Bacteriology, Capital Institute of Pediatrics, Beijing, China; Department of Bacteriology, Capital Institute of Pediatrics, Beijing, China; Department of Bacteriology, Capital Institute of Pediatrics, Beijing, China; Department of Bacteriology, Capital Institute of Pediatrics, Beijing, China; Department of Bacteriology, Capital Institute of Pediatrics, Beijing, China

## Abstract

**Objectives:**

To analyse the *in vitro* antimicrobial susceptibility of *Mycoplasma pneumoniae* (*M. pneumoniae*) isolates from 10 different provinces/municipalities across China during the 2023 epidemic.

**Methods:**

We collected respiratory specimens from paediatric patients diagnosed with *M. pneumoniae* pneumonia from 10 different provinces/municipalities including Beijing, Shanghai, Liaoning, Gansu, Shanxi, Henan, Hunan, Jiangsu, Guangxi and Guangdong across China from October to December 2023. *M. pneumoniae* was isolated and cultured from these specimens. Macrolide resistance-associated mutations A2063G or A2064G in the 23S rRNA gene were detected. Minimum inhibitory concentrations (MICs) of five antibiotics (erythromycin, azithromycin, tetracycline, moxifloxacin and doxycycline) were determined using PPLO broth and microdilution susceptibility tests.

**Results:**

A total of 190 *M. pneumoniae* isolates were analysed. All isolates harboured the A2063G mutation. All isolates (100%) were highly resistant to erythromycin (MIC range: 128–1024 mg/L) and azithromycin (MIC range: 4–256 mg/L), but susceptible to tetracycline (MIC range: ≤0.125–1 mg/L), doxycycline (MIC range: ≤0.125–1 mg/L) and moxifloxacin (MIC range: ≤0.125 mg/L). For azithromycin and tetracycline, *M. pneumoniae* isolates from different regions of China showed different susceptibilities. The MICs of tetracycline against *M. pneumoniae* from northern China were significantly lower than those from central China and southern China. Comparisons among provinces/municipalities suggested that isolates from Hunan and Beijing were more resistant to azithromycin. For tetracycline, the MICs were significantly lower among isolates from Beijing.

**Conclusions:**

During the 2023 epidemic, isolates of *M. pneumoniae* were highly resistant to macrolides but remained susceptible to tetracycline and quinolones. For the treatment of macrolide-resistant *M. pneumoniae*, tetracycline, moxifloxacin and doxycycline would be the recommended antibiotics based on our findings.

## Introduction

*Mycoplasma pneumoniae* (*M. pneumoniae*) is an important pathogen causing upper and lower respiratory infections in children and adolescents and is responsible for up to 40% of cases of community-acquired pneumonia, with higher rates during epidemic years.^1,2^ Most *M. pneumoniae* infections display as a mild respiratory illness sometimes referred as ‘walking pneumonia’, but some patients experience severe pneumonia that requires hospitalization.^3,4^

Macrolides have been used as the empirical treatment for *M. pneumoniae* infections for many years, especially in children, for whom tetracyclines and fluoroquinolones were avoided because of their potential toxicities.^5^ Okazaki *et al*.^6^ were the first to report point mutations in the 23S rRNA gene associated with macrolide resistance in *M. pneumoniae.* Macrolide-resistant *M. pneumoniae* (MRMP) has subsequently spread through Asia and eventually to Europe and North America, becoming a global health problem.^5^ The proportion of MRMP infections has shown an increasing trend worldwide, rising from 18.2% in 2000 to 76.5% in 2019. The incidence of MRMP infections was highest in Western Pacific regions, particularly in China, followed by South East Asia, the Americas and Europe.^7^ In studies published recently, the incidence of MRMP was less than 2% in Denmark in 2023, 1.9% from 2016 to 2020 in Switzerland, 7.1% from 2023 to 2024 in the USA, 24.6% from 2019 to 2020 in Japan and 100% in China in 2023, with A2063G and A2064G mutations detected in 96.6%–100% of macrolide-resistant isolates in China, Japan and Switzerland.^3,8–11^

MRMP can complicate treatment and result in ineffective antimicrobial therapy, which in turn can lead to the progression of pneumonia or other extrapulmonary complications.^1^ Infection with MRMP can lead to prolonged fever duration and hospital stays, higher incidence of extrapulmonary complications, elevated need for intensive care unit admission, longer antimicrobial therapy courses and an increased proportion of patients changed to alternative antibiotics compared with macrolide-susceptible *M. pneumoniae* infection.^2,12,13^

In late 2023, steep rises of *M. pneumoniae* infections occurred in many counties, including China, Denmark, Spain, the USA and the Netherlands.^3,10,14–16^ Macrolides including azithromycin and erythromycin were still recommended as first-line antibiotics in treating *M. pneumoniae* infections in China in 2023, with tetracyclines and fluoroquinolones such as doxycycline, minocycline, levofloxacin and moxifloxacin considered as alternatives.^17^ However, in 2023, macrolide resistance-associated mutations were detected in 100% of *M. pneumoniae* isolates in Beijing, the proportion of severe *M. pneumoniae* pneumonia cases reached 56.2%, and 100% of the azithromycin-treated patients remained febrile without significant improvements at 72 h post-azithromycin administration, posing serious challenges to treatment.^8^

Due to the slow growth and fastidious growth condition requirements of *M. pneumoniae*, the routine culture and phenotypic testing of antimicrobial drug susceptibility are impractical for clinical use, thus most antimicrobial resistance results are derived from *M. pneumoniae* molecular detection, not from clinical isolates. As a result, clinicians have little information regarding the antimicrobial susceptibility of *M. pneumoniae* on which to devise therapeutic strategies.^2^ To evaluate the drug susceptibility of *M. pneumoniae* in China more accurately, we analysed the antimicrobial susceptibility of clinical isolates of *M. pneumoniae* from 10 provinces/municipalities in late 2023, with the aim of providing valuable information to guide the selection of antibiotics of clinical therapy.

## Materials and methods

### Specimen collection and detection of *M. pneumoniae*

We collected respiratory specimens including sputum, nasopharyngeal swabs and bronchoalveolar lavage fluid from paediatric patients diagnosed with *M. pneumoniae* pneumonia in 10 provinces/municipalities throughout China from October to December 2023. These provinces/municipalities included Beijing, Shanghai, Liaoning, Gansu, Shanxi, Henan, Hunan, Jiangsu, Guangxi and Guangdong. This study was approved by the research board of the Ethics Committee of the Capital Institute of Pediatrics in Beijing, and informed consent was obtained for the collection of all specimens. Macrolide resistance-associated mutations A2063G or A2064G were detected using *M. pneumoniae* and Macrolide-Resistant isolates Diagnostic Kit (PCR Fluorescence Probing; Mole BioScience Co., Ltd, Jiangsu, China), in accordance with the manufacturer’s instructions. To distinguish between mutations at sites 2063 and 2064, PCR and sequencing was used as previously described.^18^

### Isolation of *M. pneumoniae*

Specimens were cultivated to obtain *M. pneumoniae* isolates. Difco™ PPLO Broth (Becton, Dicknson and Company, Sparks, USA) supplemented with 1% glucose (Solarbio, Beijing, China), 20% foetal bovine serum (Lanzhou Minhai Bio-Engineering Co., Ltd, Lanzhou, China), 25% yeast extract and 0.00125% phenol red (Sigma-Aldrich, CA, USA) was used for isolation.

### Antimicrobial susceptibility testing

Minimum inhibitory concentrations (MICs) for five antibiotics including erythromycin (Sigma-Aldrich, CA, USA), azithromycin (MedChemExpress, Shanghai, China), tetracycline (MedChemExpress, Shanghai, China), moxifloxacin (Sigma-Aldrich, CA, USA) and doxycycline (MedChemExpress, Shanghai, China) were determined using PPLO broth based on the broth microdilution susceptibility test. Each antibiotic concentration was set from 0.125 to 1024 mg/L as described in Clinical and Laboratory Standards Institute guidelines (CSLI) M43-A (2011 version).^19^ Medium containing 10^4^–10^5^ colony-forming units of *M. pneumoniae* was added to 96-well microplates and incubated at 37°C for 6–8 days. The MIC was defined as the lowest concentration of antimicrobial agent that inhibits the metabolism of *M. pneumoniae*, as indicated by the lack of colour change as soon as the drug-free control first exhibiting a colour change. Reference strain *M. pneumoniae* M129 (ATCC 29342) was used as the macrolide-susceptible control. Each antimicrobial susceptibility test was performed in triplicate. We interpreted the results of antimicrobial susceptibility testing according to Clinical and Laboratory Standards Institute guidelines (CLSI) M43-A (2011 version). The interpretive criteria are as follows: isolates exhibiting susceptibility to erythromycin and azithromycin demonstrate MICs of ≤0.5 mg/L, whereas resistant isolates show MICs of ≥1 mg/L. For tetracycline, doxycycline and moxifloxacin, the susceptibility breakpoints are MIC ≤2, ≤2 and ≤0.5 mg/L, respectively.

### Statistical analysis

SPSS 26.0 software (IBM, Armonk, NY, USA) was used for statistical analysis. MICs among the groups were compared using Kruskal–Wallis or Mann–Whitney *U*-test. A *P* value of <0.05 was considered to indicate statistical significance.

## Results

### Specimen collection

In total, we collected 789 specimens from paediatric patients diagnosed with respiratory tract infection, including 20 from Shanghai, 30 from Shanxi, 37 from Guangdong, 56 from Hunan, 61 from Gansu, 62 from Liaoning, 128 from Guangxi, 128 from Henan, 131 from Beijing and 136 from Jiangsu, with each sample corresponding to one patient.

### Macrolide resistance-associated mutations and *in vitro* antimicrobial susceptibility of all *M. pneumoniae* isolates

In total, 190 clinical isolates of *M. pneumoniae* were obtained in this study, including 17 from Hunan, 31 from Henan, 14 from Shanxi, 24 from Beijing, 26 from Liaoning, 6 from Shanghai, 27 from Jiangsu, 8 from Guangdong, 26 from Guangxi and 11 from Gansu. All isolates harboured the A2063G mutation. As listed in Table 1, the MICs were highest for erythromycin (MIC range: 128–1024 mg/L), followed by azithromycin (MIC range: 4–256 mg/L), and were notably lower for tetracycline (MIC range: ≤0.125–1 mg/L) and doxycycline (MIC range: ≤0.125–1 mg/L). Moxifloxacin had the best inhibitory effect, with MICs of ≤0.125 mg/L against all isolates. All isolates were resistant to erythromycin and azithromycin, but susceptible to tetracycline, doxycycline and moxifloxacin. The MIC_50_ and MIC_90_ of erythromycin were both 512 mg/L, which was 8- and 4-fold higher than those of azithromycin, respectively (Table 2). Moreover, comparisons of cumulative MIC distribution showed that the erythromycin MICs were significantly higher than the azithromycin MICs (*P* < 0.0001), revealing that *M. pneumoniae* isolates in this epidemic were more resistant to erythromycin. The MIC_50_ and MIC_90_ of doxycycline were 2-fold lower than those of tetracycline (Table 2), indicating that doxycycline may have a greater inhibitory effect against *M. pneumoniae*, and this was confirmed by the comparisons of MIC distributions (*P* < 0.0001).

**Table 1.** MIC range of different antibiotics against *M. pneumoniae* isolates from different regions across China during the 2023 epidemic

Regions		Number of isolates	MIC range (mg/L)				
			Erythromycin	Azithromycin	Tetracycline	Doxycycline	Moxifloxacin
Total		190	128–1024	4–256	≤0.125–1	≤0.125–1	≤0.125
Central China	Hunan	17	512–1024	32–256	≤0.125–1	0.25–1	≤0.125
	Henan	31	256–1024	16–256	0.25–1	≤0.125–0.5	≤0.125
	Shanxi	14	128–1024	4–128	≤0.125–1	≤0.125–0.5	≤0.125
	Total	62	128–1024	4–256	≤0.125–1	≤0.125–1	≤0.125
Northern China	Beijing	24	256–1024	16–256	≤0.125–1	≤0.125–1	≤0.125
	Liaoning	26	256–1024	8–256	≤0.125–1	≤0.125–1	≤0.125
	Total	50	256–1024	8–256	≤0.125–1	≤0.125–1	≤0.125
Eastern China	Shanghai	6	256–1024	64–128	≤0.125–0.5	≤0.125–0.25	≤0.125
	Jiangsu	27	256–1024	16–256	0.25–1	≤0.125–0.5	≤0.125
	Total	33	256–1024	16–256	0.25–1	≤0.125–0.5	≤0.125
Southern China	Guangdong	8	256–512	32–256	0.25–1	0.25–0.5	≤0.125
	Guangxi	26	256–512	8–256	0.25–1	≤0.125–1	≤0.125
	Total	34	256–512	8–256	0.25–1	≤0.125–1	≤0.125
Western China	Gansu	11	256–1024	8–256	0.25–1	≤0.125–1	≤0.125
	Total	11	256–1024	8–256	0.25–1	≤0.125–1	≤0.125

**Table 2.** MIC_50_/MIC_90_ of antibiotics against *M. pneumoniae* isolates from different regions across China during the 2023 epidemic

Regions		Number of isolates	MIC_50_/MIC_90_ (mg/L)				
			Erythromycin	Azithromycin	Tetracycline	Doxycycline	Moxifloxacin
Total		190	512/512	64/128	0.5/1	0.25/0.5	≤0.125/≤0.125
Central China	Hunan	17	512/512	128/256	1/1	0.5/1	≤0.125/≤0.125
	Henan	31	512/512	64/128	0.5/1	0.25/0.5	≤0.125/≤0.125
	Shanxi	14	512/512	32/128	0.5/0.5	0.25/0.5	≤0.125/≤0.125
	Total	62	512/512	64/128	0.5/1	0.25/0.5	≤0.125/≤0.125
Northern China	Beijing	24	512/1024	128/256	0.25/0.5	0.25/0.5	≤0.125/≤0.125
	Liaoning	26	512/512	64/128	0.5/0.5	0.25/0.5	≤0.125/≤0.125
	Total	50	512/1024	64/256	0.5/0.5	0.25/0.5	≤0.125/≤0.125
Eastern China	Shanghai	6	512/512	128/128	0.5/0.5	0.25/0.25	≤0.125/≤0.125
	Jiangsu	27	512/512	64/128	0.5/1	0.5/0.5	≤0.125/≤0.125
	Total	33	512/512	64/128	0.5/1	0.25/0.5	≤0.125/≤0.125
Southern China	Guangdong	8	512/512	64/128	0.5/1	0.5/0.5	≤0.125/≤0.125
	Guangxi	26	512/512	64/128	0.5/1	0.25/0.5	≤0.125/≤0.125
	Total	34	512/512	64/128	0.5/1	0.5/0.5	≤0.125/≤0.125
Western China	Gansu	11	512/1024	64/128	0.5/0.5	0.25/0.5	≤0.125/≤0.125
	Total	11	512/1024	64/128	0.5/0.5	0.25/0.5	≤0.125/≤0.125

### Differences in antimicrobial susceptibilities among *M. pneumoniae* isolates from five different regions in China

**Figure 1.**
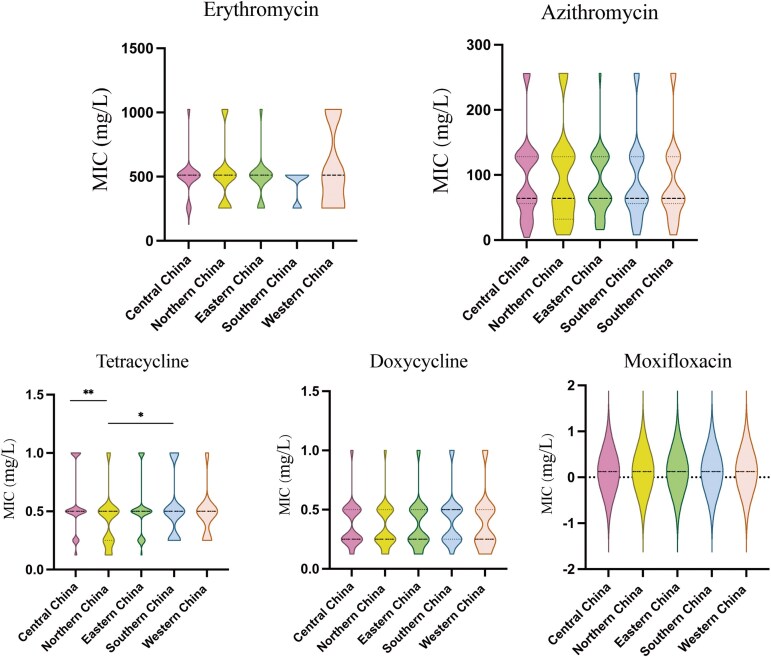
Comparisons of the minimum inhibitory concentrations (MICs) of five antibiotics against *M. pneumoniae* isolates from five different regions across China during the 2023 epidemic. *P < 0.05, **P < 0.01, ***P < 0.001, ****P < 0.0001.

To compare the antibiotic susceptibilities of *M. pneumoniae* isolates from different regions, we combined some provinces/municipalities and regrouped them according to their locations, dividing them into eastern, western, southern, northern and central China. Isolates from these five different regions showed different susceptibilities for tetracycline (*P* = 0.0387). The MIC_50_ of tetracycline was 0.5 mg/L in all regions, with a relatively higher MIC_90_ in central, southern and eastern China (Table 2). Comparisons of MIC distributions showed that tetracycline MICs were significantly lower in northern China than central China (*P* = 0.0027) and southern China (*P* = 0.0477, Figure 1).

As listed in Table 2, the MIC_50_ of erythromycin was the same for isolates from the five different regions, at 512 mg/L; similarly, the MIC_50_ of azithromycin was 64 mg/L for isolates from all regions. The MIC_90_ value of erythromycin was highest in northern and western China. The MIC_90_ of azithromycin was highest in northern China. The MIC_50_ of doxycycline was 0.5 mg/L in southern China and 0.25 mg/L in the other regions, with an MIC_90_ of 0.5 mg/L in all regions. However, no significant difference was found in the MIC distributions of erythromycin (*P* = 0.7386), azithromycin (*P* = 0.9629) and doxycycline (*P* = 0.6596, Figure 1).

### Differences in antimicrobial susceptibilities among *M. pneumoniae* isolates from 10 different provinces/municipalities in China

**Figure 2.**
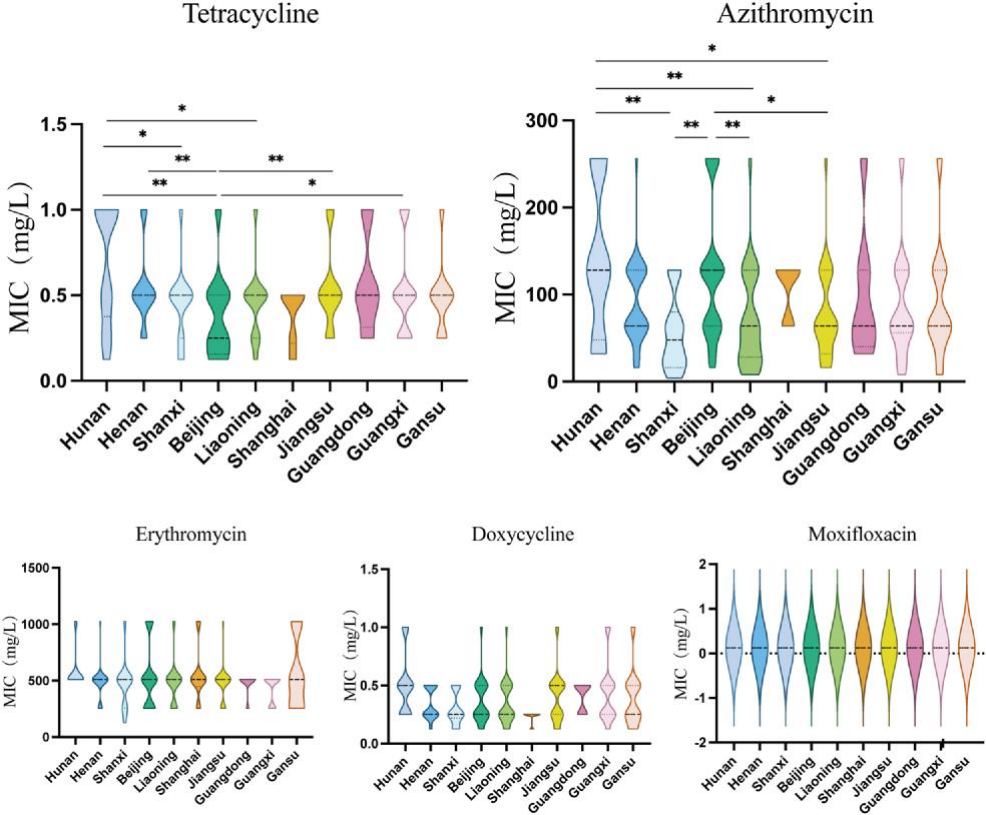
Comparisons of the minimum inhibitory concentrations (MICs) of five antibiotics against *M. pneumoniae* isolates from 10 different provinces/municipalities across China during the 2023 epidemic. *P < 0.05, **P < 0.01, ***P < 0.001, ****P < 0.0001.

*M. pneumoniae* isolates from different provinces/municipalities exhibited significantly different susceptibilities for azithromycin (*P* = 0.011) and tetracycline (*P* = 0.035). The MIC_50_ of azithromycin was highest against isolates from Beijing, Hunan and Shanghai (128 mg/L). The MIC_90_ of azithromycin was 256 mg/L against isolates from Beijing and Hunan, and 128 mg/L in the other provinces/municipalities (Table 2). Comparisons showed that MICs were significantly higher in isolates from Beijing and Hunan than those from Jiangsu (*P* = 0.0251, *P* = 0.0189), Shanxi (*P* = 0.0039, *P* = 0.0035) and Liaoning (*P* = 0.0081, *P* = 0.0062) (Figure 2). These results suggested that during the 2023 epidemic, *M. pneumoniae* isolates in Hunan and Beijing were more resistant to azithromycin.

For tetracycline, the MIC_50_ was 0.25 mg/L against isolates from Beijing, 1 mg/L against isolates from Hunan and 0.5 mg/L against isolates from the other seven provinces/municipalities and the MIC_90_ was 0.5 mg/L against isolates from Beijing, Shanxi, Liaoning, Shanghai and Gansu and 1 mg/L against isolates from the remaining five provinces/municipalities (Table 2). Comparisons showed that MICs of tetracycline were significantly lower in isolates from Beijing compared with those from Hunan (*P* = 0.0033), Guangxi (*P* = 0.0269), Jiangsu (*P* = 0.0088) and Henan (*P* = 0.0028) (Figure 2).

For erythromycin, the MIC_50_ was 512 mg/L for all isolates, whereas the MIC_90_ was higher in Beijing and Gansu (Table 2). For doxycycline, the MIC_50_ was 0.5 mg/L in Hunan, Jiangsu and Guangdong, and 0.25 mg/L in the other provinces/municipalities, whereas the MIC_90_ was highest in Hunan (1 mg/L), lowest in Shanghai (0.25 mg/L) and 0.5 mg/L in the other provinces/municipalities (Table 2). However, there was no significant difference in the MIC distributions among different provinces/municipalities for these two antibiotics.

## Discussion

*M. pneumoniae* is a common pathogenic cause of respiratory infections in children, causing epidemics with a recognized cycle interval of 3–7 years.^20^ The most recent worldwide epidemic occurred in late 2023.^3,8,10^

*M. pneumoniae* is inherently resistant to beta-lactams, glycopeptides and fosfomycin antimicrobials due to the lack of a cell wall. The main drug classes with efficacy against *M. pneumoniae* are agents acting on bacterial ribosomes to inhibit protein synthesis, including macrolides, ketolides, streptogramins and tetracyclines, as well as agents that inhibit DNA replication, such as the fluoroquinolones.^5^ However, tetracyclines could bring adverse effects including enamel hypoplasia, permanent grey/brown discolouration of teeth and temporary bone growth inhibition in children; fluoroquinolones can lead to cartilage damage and tendon rupture; thus, the use of tetracyclines and fluoroquinolones is restricted in children under 8 years of age and in patients under 18 years of age, respectively.^17,21^ Macrolides are safe and well-tolerated in oral formulations, and possess anti-inflammatory properties independent of their anti-bacterial activity.^22^ They used to be one of the most potent agents against *M. pneumoniae*.^5^ These advantages made macrolides the empirical antibiotics of choice in treating infections caused by *M. pneumoniae* for many years. However, the excessive and inappropriate use of macrolides resulted in the emergence of MRMP.^23^ As evidenced by *in vitro* antimicrobial susceptibility tests, the resistance of *M. pneumoniae* to macrolides has been increasing in China in recent years. Zhao *et al*.^24^ reported that in Beijing from 2014 to 2016, 65.4% (53/81) of *M. pneumoniae* isolates showed resistance to erythromycin and azithromycin, with an MIC range of ≥256 and 2–64 mg/L, respectively. In 2018, among 154 *M. pneumoniae* isolates from five cities of China, 79.9% were resistant to erythromycin (MIC range: 128–>256 mg/L) and azithromycin (MIC range: 2–32 mg/L).^20^ From 2017 to 2019 in Shanghai, 97.3% (177/182) of *M. pneumoniae* isolates were resistant to erythromycin (MIC: ≥64 mg/L), and the macrolide resistance rate increased from 85.7% in 2017 to 100% in 2019.^25^ In our study, 100% of *M. pneumoniae* isolates were resistant to macrolides erythromycin and azithromycin, revealing a serious issue regarding macrolide resistance across China. In particular, the maximum MIC for azithromycin has increased from 64 mg/L in 2014–19 to 256 mg/L in 2024, and the MIC_50_ and MIC_90_ values for azithromycin in our study were higher than those in Shanghai from 2017 to 2019 (MIC_50_: 16 mg/L; MIC_90_: 32 mg/L), suggesting that *M. pneumoniae* has become more resistant to azithromycin in China.

*M. pneumoniae* isolates resistant to tetracyclines and fluoroquinolones have not been detected until now, and MICs for these two antibiotics remain low. From 2014 to 2018 in China, the MIC ranges for tetracycline and levofloxacin were 0.016–0.5 and 0.125–1 mg/L, respectively.^20,24^ From 2017 to 2019 in Shanghai, the MIC ranges for tetracycline, doxycycline and moxifloxacin were 0.06–2, 0.015–1 and 0.015–0.25 mg/L, and the MIC_50_/MIC_90_ values for each antibiotic were 0.5/1, 0.25/0.5 and 0.125/0.25 mg/L, respectively.^25^ Tetracyclines and fluoroquinolones are recommended as alternative antibiotics for the treatment of *M. pneumoniae* infection.^17,26^ Currently, for MRMP pneumonia treatment, newer tetracyclines, including doxycycline and minocycline, are recommended, and fluoroquinolones are considered as second-line drugs, based on careful consideration of the risks and benefits, along with parental informed consent.^1^ Clinical data have demonstrated that tetracyclines and fluoroquinolones have greater efficacy in treating MRMP infections, patients treated with tetracyclines or fluoroquinolones had shorter fever duration and hospital stays compared with those treated with macrolides.^27–29^ Furthermore, MRMP pneumonia patients treated with doxycycline had shorter times to defervescence and chest X-ray improvement compared with those treated with macrolides.^30^ A retrospective study comparing the effectiveness of minocycline and doxycycline in treating severe MRMP showed that patients who received doxycycline had significantly higher fever resolution rates within 24, 48 and 72 h compared with minocycline.^31^ In our study, *M. pneumoniae* isolates collected in China in 2023 were all susceptible to tetracycline, doxycycline and moxifloxacin, with MICs lower than 1 mg/L. Collectively, these results showed that tetracycline, doxycycline and moxifloxacin could be considered as recommended antibiotics in treating MRMP infections.

Several limitations exist in our study. First, we used PPLO broth for antimicrobial susceptibility testing while Clinical and Laboratory Standards Institute guidelines (CSLI) M43-A (2011 version) recommend SP4 broth. Second, the number of *M. pneumoniae* isolates was unevenly distributed both quantitatively and regionally, being relatively lower in some regions due to the insufficient availability of clinical specimens, and only one city in western China was included in our study. Third, our study reflects the inhibitory effect of antibiotics against *M. pneumoniae in vitro*, the inhibitory effect *in vivo* is unclear. Thus, future studies may additionally investigate clinical data including patient’s symptoms, antibiotic treatment records and clinical outcomes in China during the 2023 epidemic.

### Conclusion

During the 2023 epidemic, all *M. pneumoniae* isolates across China were highly resistant to macrolides but remained susceptible to tetracyclines and fluoroquinolones. Resistance to azithromycin has increased among *M. pneumoniae* isolates compared with isolates reported in previous years. For azithromycin and tetracycline, isolates from different regions of China showed different susceptibilities, but these differences did not affect clinical medication, since all isolates were resistant to azithromycin and susceptible to tetracycline. For the treatment of MRMP, tetracycline, moxifloxacin and doxycycline could now be considered the recommended antibiotics.
